# Evaluation of Antidiarrheal Activity of 80% Methanol Extract and Solvent Fractions of the Leaves of *Withania somnifera* (L.) Dunal in Swiss Albino Mice

**DOI:** 10.1155/2022/7968973

**Published:** 2022-05-09

**Authors:** Getaye Tessema Desta, Yared Andargie Ferede, Woretaw Sisay Zewdu, Muluken Adela Alemu

**Affiliations:** School of Pharmacy, College of Health Sciences, Debre Tabor University, P.O. Box 272, Debre Tabor, Ethiopia

## Abstract

**Background:**

*Withaniasomnifer*a is an important medicinal plant for the treatment of diarrhea in Ethiopian folklore medicine. The aim of this study was to evaluate the antidiarrheal activity of *Withania somnifera* leaves in *Swiss* albino mice.

**Materials and Methods:**

Hydromethanolic crude extraction and solvent fractionation were done using cold maceration technique. 80% methanol was used as a solvent in crude extraction, while distilled water, n-butanol, and chloroform were employed during fractionation. Castor oil-induced diarrhea, enteropooling, and gastrointestinal motility models were employed to evaluate antidiarrheal activity. Mice were randomly divided into five groups (six mice per group): negative control, which received 2% Tween 80 in distilled water; positive control, which received 3 mg/kg loperamide; and three test groups (III, IV, and V), which were treated with 100 mg/kg, 200 mg/kg, and 400 mg/kg of crude extract and solvent fractions, respectively.

**Results:**

The crude extract, aqueous, and n-butanol fractions significantly delayed the onset of diarrhea at 200 mg/kg and 400 mg/kg dose. There was a significant reduction in the number and weight of stools at all tested doses of the crude extract and aqueous fraction, and at 200 mg/kg and 400 mg/kg of n-butanol fraction. Significant reduction in volume and weight of intestinal contents was observed at all tested doses of the crude extract, and at 200 mg/kg and 400 mg/kg of aqueous and n-butanol fractions. All tested doses of the crude extract and 200 mg/kg and 400 mg/kg of the aqueous and n-butanol fractions significantly reduced the motility of charcoal meal.

**Conclusion:**

This study demonstrated that the crude extract and solvent fractions of the *Withania somnifera* leaves have antidiarrheal activity and supported the folklore use of the plant.

## 1. Background

Diarrhea is a common disorder of the gastrointestinal system that affects all age groups universally. The term diarrhea was originated from the Greek word “dia” (through) and Latin word “rhein” (flow), which denote the flow of intestinal contents through the bowel of the gastrointestinal tract [1]. Although the definition of diarrhea is subjective, it is generally defined as the discharge of voluminous unformed (loose) stools more frequently than the normal (usually more than three times per day). Clinically, the patient complains about the reduction of stool consistency, increase in the weight of stool, and urgent defecation [2]. Pathologically, diarrhea is caused as a result of the imbalance between fluid secretion and absorption capacity of the intestine lumen, in which there is overstimulation of fluid secretion and reduction of fluid absorption capacity of the intestinal lumen. Diarrhea can be classified in different ways. In terms of frequency of symptoms, it can be acute, persistent, and chronic. Acute diarrhea is the passage of fluidly unformed stool more than three times per day for less than one week and is mostly self-limited but causes severe dehydration. Persistent diarrheal is defined as the presence of diarrhea symptoms from two to four weeks, while in chronic diarrhea, the symptoms of diarrhea last for more than a month and mostly require administration of antidiarrheal medication [3]. On the basis of pathophysiologic mechanism, it can be secretary diarrhea, exudative diarrhea, osmotic diarrhea, and motility-related diarrhea [4]. Diarrhea affects all regions of the world, but it is highest in developing countries, particularly sub-Saharan Africa and South Asia. It has many impacts such as lowering the quality of life, prolonging hospitalization stays, increasing the cost of the healthcare system, and causing deaths [5]. Epidemiological studies revealed that diarrhea had been the second cause of death, next to pneumonia, in under-five children globally by 2021 [6]. Diarrhea-related child death was found at a higher magnitude (78%) in the disadvantaged regions such as African and Southeast Asian [7].

Diarrhea can be treated using conventional antidiarrheal agents and traditional medicine practice. The conventional antidiarrheal drugs diminish the symptoms and signs of diarrhea via improving the consistency of stool, decreasing the frequency of defecation, and/or reducing the weight of stool. Antidiarrheal drugs mediate their action through inhibition of intestinal fluid secretions and motility of intestinal segment and enhancing absorption capacity of the intestinal lumen [8]. However, these conventional drugs are not easily accessible to the majority of people particularly in developing countries with affordable prices; cause terrible side effects; develop addiction during chronic treatment such as opioids; lack curative action during oral rehydration therapy; and are contraindicated such as loperamide for acute ulcerative colitis, patients younger than two years, and bloody diarrhea [9, 10]. On the contrary, plants are easily accessible with affordable prices, with more cultural acceptance by a vast majority of people. Consequently, the majority of people particularly in developing countries preferably utilize plant remedies for the management of diarrhea. Therefore, searching for and manufacturing safe, effective, and cheap antidiarrheal drugs constitute an imperative issue for health professionals and pharmaceutical companies. According to World Health Organization (WHO) report, 80% of people still depend on the use of plants for the treatment of various diseases including diarrhea, and 25% of drugs were discovered from plant sources which were used in the traditional medicine practice [11]. Ethiopia is a developing country, where 80% of the people and 90% of the livestock depend on plants for the prevention, cure, and treatment of numerous diseases [12]. Among these diseases, diarrhea is the main one that is treated using plant remedies. In Ethiopian traditional medicine practices, many plants have been acclaimed for the management of diarrhea. Among these plants, the roots of *Rumex nepalensis* Spreng.; the seeds and leaves of *Ruta chalepensis* L.; the roots of *Solanum incanum* L. [13]; the seeds of *Linum usitatissimum* L.; the roots of *Salvia nilotica* Jacq. [14]; and the leaves of *Verbena Officinalis* L., *Vernonia amygdalina* Del., and *Withania somnifera* (L.) Dunal [15] are frequently used.

*Withania somnifera* (*W. somnifera*) is classified under the family Solanaceae and is native to India. It is an erected, branched evergreen shrub that is 30–150 cm in length. The leaves are simple, glabrous, and ovate and elongate up to 10 cm in height with greenish or lurid yellow flowers. Globally, it has been distributed from Southern Mediterranean regions to the Canary Island; from Palestine to North India covering Israel, Jordan, Egypt, Sudan, Iran, Baluchistan, Afghanistan, and Pakistan; and from South to East Africa including Ethiopia [16, 17]. In Ethiopia, *W. somnifera* is distributed throughout the region and known with different local names such as “Giziewa” in Amharic [18], “Lallaafaa” in Afaan Oromoo [19], and “Agol” in Tigray [20]. In Ethiopian traditional medicine practices, the leaves of *W. somnifera* have been acclaimed for the treatment of diarrhea [15, 21–23]. In the acclaimed area, the people extract the dried or fresh leaf of the plant through maceration and infusion techiniques using water as a solvent, and then they drink the filtrate. Beyond the use for diarrheal treatment, different parts of the *W. somnifera* are also used for treatment of different diseases. For instance, the leaves and the roots have been used for the treatment of spirit, evil eye, swelling, and gallstone [20, 24], and the leaves have been used for the treatment of febrile illness [15]. Furthermore, numerous *in vitro* and *in vivo* tests were done on different parts of *W. somnifera*. The leaves of *W. somnifera* showed anti-inflammatory activity against stainless steel implant-induced inflammation in adult zebra fish [25]; anxiolytic and anti-neuroinflammatory activity against systemic lipopolysaccharide-induced neuroinflammation in albino rats and in *in vitro* test [26, 27]; antimalarial activity in *Plasmodium berghei* infected mice [28]; antidiabetic and antihyperlipidemic activity in alloxan-induced diabetic rats [29, 30]; cyclooxygenase-2 (COX-2) enzyme inhibition [31]; and anti-genotoxicity, antioxidant, and anticancer effects against hepatocellular carcinoma cell line [32]. The qualitative phytochemical screening test also revealed the existence of tannins, cardiac glycosides, steroids, reducing sugars, flavonoids, saponins, phenols, and alkaloids [33, 34]. The root extract also demonstrated antioxidant activity, free radical scavenging activity, and anti-inflammatory activity against collagen-induced arthritic and carrageenan-induced paw edema in rats [35]. An *in vitro* study on the ethanol root extract demonstrated enzyme inhibitory activity on cyclooxygenases and lipoxygenase and antidiabetic activity against alloxan-induced diabetes in rats [30, 36]. Extract from the whole plant also showed bronchodilator, vasodilator, and diuretic activity [37]. Withanolides isolated from *W. somnifera* revealed a dose-dependent spasmolytic and Ca^2+^ antagonistic activity on spontaneously contracting jejunum isolated from rabbit jejunum [38]. All this information and the traditional claims were triggers to do this experiment. Furthermore, up to this moment, there was no *in vivo* antidiarrheal activity test on the leaves of the plant. Hence, this experiment aimed to evaluate the antidiarrheal activity of 80% methanol crude extract and solvent fractions of the leaves of *W. somnifera* in *Swiss* albino mice.

## 2. Materials and Methods

### 2.1. Drugs and Chemicals

To conduct this experiment, the following drugs and chemicals were used: distilled water, loperamide hydrochloride (Remedica, Cyprus), castor oil (Amman Pharmaceutical Industries, Jordan), absolute methanol (Carlo Erba Reagents, SAS, France), chloroform (Hi-Media Laboratory Reagents, India), n-butanol (Blulux Laboratories Ltd., India), activated charcoal (Acurouate Organics Ltd., New Delhi), Mayer's reagent and ethyl acetate (May and Baker Ltd., Dagenham, England), ferric chloride (BDH Ltd., England), potassium ferrocyanide (BDH Ltd., England), ammonia (Merck Millipore, India), acetic anhydride (Techno Pharmchem, India), lead acetate test (Fisher Scientific, UK), and sulfuric acid (Farmitalia Carlo Erba, Italy).

### 2.2. Plant Materials

The fresh leaves of *W. somnifera* were collected from the Debre Tabor district, Amhara region, situated about 97 km from the Bahir Dar city and 667 km from Addis Ababa, northwest Ethiopia, in September 2021. The plant specimen was identified and authenticated by a botanist from the Biology Department, College of Natural and Computational Sciences, Debre Tabor University, with the specimen number GT-024 and deposited for future reference. The fresh leaves were washed using tap water and dried at room temperature under shade for two weeks in the pharmacy laboratory room. The dried leaves were milled into a coarse powder using mortar and pestle and stored in a clean plastic container until extraction.

### 2.3. Extraction of the Plant Materials

#### 2.3.1. Extraction of the Crude Extract

Cold maceration technique and eighty percent (80%) methanol solvent were used for crude extraction. Seven hundred gram (700 g) of leaves was macerated in 3500 ml of 80% methanol for three consecutive days in Erlenmeyer conical flasks with occasional stirring and agitation. After three days of maceration, the extract was filtered through a double-layer muslin cloth, followed by Whatman No. 1 filter paper. The marc remaining after separation was re-macerated and filtered twice by adding fresh 80% methanol in the same manner as the previous one. The filtrates from each successive maceration were mixed up together, and methanol was removed from the filtrate by evaporation under vacuum in a rotary evaporator (Yamato, Japan) at 40°C. Then, the aqueous residue was removed by deep freezing after being stored overnight and lyophilized through a lyophilizer (Operon, Korea Vacuum Limited, Korea). The obtained extract was stored in an airtight bottle in a refrigerator until it was subjected to further solvent fractionation and experimental procedure. The fresh stock solution was prepared just before the actual experiment was done with 2% Tween 80 in distilled water.

#### 2.3.2. Preparation of Solvent Fractions

The solvent fractions were obtained using chloroform, n-butanol, and distilled water in increasing order of their polarity. The fractionation was done according to the method described by Mengie et al. [39]. Eighty gram (80 g) of 80% methanol crude extract of *W. somnifera* was dissolved in 400 ml of distilled water in a separator funnel. An equal volume of chloroform was added to the aqueous solution, and they were mixed well by gentle agitation. Then, the mixture was allowed to form a distinct layer (the chloroform at the bottom and the aqueous solution at the top). After 24 h, the chloroform fraction was isolated from the mixture, and the process was repeated twice with fresh chloroform. All the chloroform fractions were collected together and subjected to evaporation via a Rotary Evaporator (Yamato, Japan), which was set at 40°C to obtain the chloroform fraction. The remaining aqueous residue was further fractionated with 400 ml of n-butanol in the same manner as chloroform. The remaining aqueous residue after removal of n-butanol was frozen in a deep freezer overnight. Then, the frozen residue was dried with a lyophilizer (Operon, Korea Vacuum Limited, Korea). The three fractions were labeled and kept in the deep freezer with airtight containers in the refrigerator at −20°C until they were used in the antidiarrheal test.

### 2.4. Animal Grouping and Dosing

The experiment was conducted using either sex of healthy Swiss albino mice having 6–8 weeks of age and weights of 20–30 g. The mice were purchased from the Ethiopian Health and Nutrition Research Institute (EHNRI), Addis Ababa, Ethiopia, and kept in a clean cage under a standard laboratory condition with a 12 h light-dark cycle. The mice were allowed free access to standard pellet food and water. Before the experiment was run, the mice were acclimatized for 14 days in working laboratory. At the time of the experiment, the mice were randomly categorized into five groups with six mice per group. The groups were allocated as group I (negative control) receiving 10 ml/kg 2% Tween 80 in distilled water; group II (positive control) receiving 3 mg/kg loperamide; and group III to group V (test groups) receiving 100, 200, and 400 mg/kg doses of the crude extract and solvent fractions, respectively. All mice were administered their respective treatment dose orally using oral gavage. During running the experiment, the mice were handled according to the international standard guidelines set for the Care and Use of Laboratory Animals [40].

### 2.5. Preliminary Phytochemical Screening

The phytochemical constituents of the crude extract and solvent fractions were evaluated for the presence or absence of tannins, saponins, flavonoids, terpenoids, alkaloids, glycosides, steroids, phenols, and anthraquinones according to the methods described [41, 42].

### 2.6. Acute Toxicity Study

An oral acute toxicity test was done for the crude extract according to the limit test recommendation of the Organization for Economic Cooperation and Development (OECD) guideline 425 [43]. Five healthy 8- to 12-week-old nulliparous and nonpregnant young female mice were randomly selected and acclimatized for five days before the actual experiment conducted. The mice were deprived from food but allowed access to water for 3 h, and they were weighed just before dosing the extract. After dosing the extract, the mice were deprived from food for 1 h, but access to water was unrestricted. On the initial day of the test, a single mouse received 2000 mg/kg crude extract orally using oral gavage. According to the first mouse result, the next procedure was run on the remaining four mice in a similar way to the first procedure. The mouse was followed up for any change in physiological effects on, for example, skin and fur, eyes and mucous membranes, and also respiration and behavioral manifestation every 30 minutes in the first 4 h of dosing. Then, a daily observation for a total of 14 days was continued.

## 3. Determination of Antidiarrheal Activity

### 3.1. Castor Oil-Induced Diarrhea

This model was done according to the method described earlier by Kifle et al. [44]. Swiss albino mice of either sex were fasted for 18 h from food with free access to water and were randomly allocated to five groups with six mice per group. After 1 h administration of the corresponding treatment doses, the mice received 0.5 ml of castor oil, and they were situated in a distinct plastic cage, where the floor was lined with transparent paper. The paper was altered every 1 h for a total of 4 h duration. After 4 h follow-up, the beginning of diarrhea (the time interval in minutes between the administration of castor oil and the appearance of the initial diarrheal stool), the number of the wet and total (dry and wet) stools, and the weight of the fresh stools were recorded. The percentage of diarrheal inhibition was calculated according to (1). The total number of wet stools for the control group was considered to be 100%.

(1) $$\begin{array}{c} \text{Percentage of diarrheal inibition}=\frac{\text{Mean number }of\left(\text{WSC}-\text{WFT}\right)}{\text{Mean number of WFC}}\times 100, \end{array}$$

where WSC is the wet stool of the control group and WST is the wet stool of the test group.

### 3.2. Castor Oil-Induced Enteropooling

This model was done based on the method described previously by Gudeta et al. [45]. The experimental animals were deprived of both food and water for 18 h and randomly divided into their respective groups as described earlier in Animal Grouping and Dosing. Then, the animals received their respective treatment doses, and after 1 h, the mice were administered 0.5 ml of castor oil. After 1 h, the mice were sacrificed by cervical dislocation, and the small intestine from the pylorus to the caecum was carefully separated after opening the abdomen. The small intestine was tied at the two ends and then weighed on an electrical balance. Then, the contents of the intestine were milked into a graduate tube, and the volume of the content was recorded. Thereafter, the empty intestine was weighted. The weight difference was calculated by subtracting the weight of the empty intestine from that of the full intestine, and the result was recorded. The percentage of reduction in the intestinal contents, in terms of volume and weight, was calculated relative to the negative control according to (2) and (3), respectively.

(2) $$\begin{array}{c} \text{\% of inhibition of volume of intenstinal contents}=\frac{\text{MVICC}-\text{MVICT}}{\text{MVICCC}}\times 100, \end{array}$$

where MVICC is the mean volume of intestinal content of the control group and MVICT is the mean volume of intestinal content of the test group.

(3) $$\begin{array}{c} \text{\% of inhibition of weight of intenstinal contents}=\frac{\text{MWICC}-\text{MWICT}}{\text{MWICCC}}\times 100, \end{array}$$

where MWICC is the mean weight of intestinal content of the control group and MWICT is the mean weight of intestinal content of the test group.

### 3.3. Castor Oil-Induced Gastrointestinal Motility

This experimental procedure was performed following the method described by Gudeta et al. [45]. The mice were fasted from food for 18 h but allowed access to water. Arbitrarily, they were divided into their respective groups as described in Animal Grouping and Dosing and received their corresponding doses. After 1 h administration of the dose, the mice received 0.5 ml of castor oil. Following 1 h of administration of the castor oil, the mice received 0.5 ml of 5% activated charcoal meal in distilled water. The mice were sacrificed by cervical dislocation after 30-minute administration of charcoal meal, the abdomen was opened, and the small intestine from the pylorus to caecum was carefully removed and placed on a clean table. The distance traveled by the charcoal meal from the pylorus toward the caecum and the entire length of the small intestine were measured with a ruler. The peristalsis index and percentage inhibition of intestinal motility were calculated based on (4) and (5), respectively.

(4) $$\begin{array}{c} \text{PI}=\frac{\text{Mean distance traveled by charcoal meal}}{\text{Mean length of small intestine}}\times \;100, \end{array}$$

where PI is the peristalsis index.

(5) $$\begin{array}{c} \%\text{ inhibition of intestinal motility}=\frac{\text{PI}\left(\text{negative control}–\text{test group}\right)}{\text{PI of negative control group}}\times 100, \end{array}$$

where PI is the peristalsis index.

### 3.4. In Vivo Antidiarrheal Index (ADI)

The *in vivo* antidiarrheal index of the crude extract and the solvent fractions of *W. somnifera* leaves was calculated by assembling the delay in defecation (time of onset, *Dfreq*), the gut meal travel distance (*Gmeq*), and purging frequency (*Pfreq*) in the number of the wet stools as main parameters based on the following formula [46].

(6) $$\begin{array}{c} \text{ADI}=\sqrt[{a}]{\text{Dfreq}\times \text{Gmeq}\times \text{Pfreq}}, \end{array}$$

where ADI is the *in vivo* antidiarrheal index.

(7) $$\begin{array}{c} Df\;req=\frac{\text{Onset of diarrhea in the}\left(\text{treated group}-\text{negative control group}\right)}{\text{Onset of diarrhea in the negative control group}}\times 100. \end{array}$$

*Dfreq* is the delay in defecation time or diarrhea onset obtained from castor oil-induced test and calculated based on the following formula.

*Gmeq* is the gut meal travel reduction as a percentage of negative control and is calculated based on the following formula.

(8) $$\begin{array}{c} \text{Gmeq}=\frac{\text{DTCM in the}\left(\text{negative control}-\text{test}\right)\text{group}}{\text{DTCM in the negative control group}}\times \;100, \end{array}$$

where DTCM is the distance traveled by the charcoal meal.

*Pfreq* is the reduction in purging frequency in the number of wet stools as a percentage of the negative control.

(9) $$\begin{array}{c} \text{Pfreq}=\frac{\text{MNWS of}\left(\text{negative control}-\text{test}\right)\text{group}}{\text{MNWS of negative control group}}\times 100, \end{array}$$

where MNWS is the mean number of wet stools.

## 4. Statistical Analysis

The data obtained from the experiment were analyzed using the Statistical Package for the Social Sciences (SPSS) version 24, and the results were expressed as mean ± standard error of the mean (SEM). The significant difference between the groups was analyzed via one-way ANOVA followed by Tukey's post hoc test and considered statically significant when the *p* value was less than 0.05.

## 5. Results

### 5.1. The Yield of Extraction

After the end of the extraction of the crude and the solvent fractions, the yields of *W. somnifera* leaves extractions were 122.99 g (17.57%), 36.42 g (45.53%), 21.18 g (26.48%), and 16.68 g (20.85%) for crude extract, aqueous fraction, n-butanol fraction, and chloroform fraction, respectively.

### 5.2. Preliminary Phytochemical Screening

The outcome of phytochemical screening of crude extract and solvent fractions of *W. somnifera* leaves is presented in Table 1. The crude extract of *W. somnifera* showed the occurrence of flavonoids, alkaloids, tannins, steroids, phenols, terpenoids, and saponins. When coming to the solvent fractions, the aqueous fraction showed the presence of flavonoids, tannins, saponins, and phenols. Tannins, flavonoids, steroids, and phenols were detected in the n-butanol fraction. On the other hand, the chloroform fraction revealed the presence of steroids, terpenoids, and flavonoids. The anthraquinones and glycosides were absent across the crude extract and the solvent fractions.

### 5.3. Acute Oral Toxicity Test

During the acute oral toxicity test of the crude extract, there were no deaths or signs of acute toxicity—like changes in skin and fur, eyes and mucous membranes, and respiratory and circulatory system; anxiety; polyuria; diarrhea; and seizure—during the 14-day follow-up period at 2000 mg/kg dose. According to this, the leaf extract of *W. somnifera* at 2000 mg/kg displayed a good margin of safety with the LD_50_ value greater than 2000 mg/kg.

### 5.4. The Effects on Castor Oil-Induced Diarrheal Model

The antidiarrheal effect of the crude extract of *W. somnifera* leaves on the castor oil-induced diarrheal model is shown in Table 2. As compared to the negative control group, the crude extract of *W. somnifera* leaves caused a significant delay in the onset of diarrhea at 200 mg/kg and 400 mg/kg doses, and all the three doses showed a reduction of the diarrheal stool defecation (the number of wet stools), the total number of wet and dry stools, and the weight of fresh stools. The percentage of diarrheal stool inhibition at 100 mg/kg, 200 mg/kg, and 400 mg/kg was 29.41% (*p* < 0.05), 56.86% (*p* < 0.001), and 80.39% (*p* < 0.001), respectively.

Relative to the negative control group, 200 mg/kg and 400 mg/kg doses of aqueous and n-butanol fractions showed a significant delay in the onset of diarrhea, while the chloroform fraction did only at 400 mg/kg dose. The aqueous fraction revealed a significant reduction in the frequency (in both the wet and total stools) and the weight of fresh diarrheal stools at 100 mg/kg, 200 mg/kg, and 400 mg/kg doses with a percentage of diarrhea inhibition of 23.53% (*p* < 0.05), 50.98% (*p* < 0.001), and 62.74% (*p* < 0.001), respectively. The n-butanol fraction showed a significant effect on the reduction of both the number of (wet and total) stools and the weight of fresh stools at 200 mg/kg and 400 mg/kg with a percentage of diarrheal inhibition of 27.41% (*p* < 0.01) and 60.78% (*p* < 0.001). On the other hand, the chloroform fraction only showed a statistically significant reduction in the frequency of stool (both wet and total) and the weight of fresh stool at 400 mg/kg with 45.10% (*p* < 0.001) diarrheal inhibition.

### 5.5. The Effects on Castor Oil-Induced Enteropooling

The crude extract of *W. somnifera* leaves showed a significant reduction in intraluminal fluid accumulation relative to the negative control group at all three doses. The percentage of volume reduction at 100 mg/kg, 200 mg/kg, and 400 mg/kg was 12.31% (*p* < 0.05), 36.91% (*p* < 0.001), and 54.69% (*p* < 0.001), respectively. The reduction of weight at the 100 mg/kg, 200 mg/kg, and 400 mg/kg was 7.57% (*p* < 0.05), 16.45% (*p* < 0.001), and 46.97% (*p* < 0.001), respectively. The effect of crude extract and solvent fractions of *W. somnifera* leaves on the castor oil-induced enteropooling is depicted in Table 3.

The decrease in the volume and weight of intestinal contents at 200 mg/kg and 400 mg/kg doses of aqueous and n-butanol fractions was significant relative to the negative control group. The percentage of volume reduction in aqueous fraction was 40.15% (*p* < 0.001) and 51.55% (*p* < 0.001) and for n-butanol fraction was 34.44% (*p* < 0.01) and 47.27% (*p* < 0.001) at 200 mg/kg and 400 mg/kg, respectively. Meanwhile, the percentage of reduction in weight for aqueous fractions was 44.87% (*p* < 0.001) and 49.58% (*p* < 0.001), and the n-butanol fraction was 34.62% (*p* < 0.01) and 46.15% (*p* < 0.001) at 200 mg/kg and 400 mg/kg, respectively. The chloroform fraction failed to show a significant reduction in volume and weight of the intestinal contents at 100 mg/kg and 200 mg/kg as compared to the negative control group. The percentage of reduction in volume and weight of intestinal contents at 400 mg/kg of chloroform fraction was 33.02% (*p* < 0.001) and 33.33% (*p* < 0.001), respectively.

### 5.6. The Effects on Castor Oil-Induced Gastrointestinal Motility

The result of this experiment is displayed in Table 4. The crude extract of *W. somnifera* leaves revealed a significant reduction in the movement of the charcoal meal at all the tested doses compared to the negative control. The percentage of reduction in the transition of charcoal meal at 100 mg/kg, 200 mg/kg, and 400 mg/kg was 21.69% (*p* < 0.01), 41.04% (*p* < 0.001), and 57.10% (*p* < 0.001), respectively. The result of the crude extract at the 400 mg/kg dose was nearly the same as that of the 3 mg/kg loperamide (60.89% vs. 58.39%, respectively).

There was a significant effect on the transit of charcoal meals at the middle and higher doses of the aqueous and n-butanol fractions. However, the lower dose of them failed to reveal a significant effect on the transit of charcoal meals as compared to the negative control group. The percentage of reduction in the intestinal motility of the charcoal at the middle and higher doses was 38.32% (*p* < 0.001) and 53.14% (*p* < 0.001) for aqueous fraction and 25.79% (*p* < 0.001) and 41.54% (*p* < 0.001) for n-butanol fraction, respectively. The percentage of reduction in the transit of charcoal meal at 400 mg/kg of chloroform fraction was 29.82% (*p* < 0.001). The lower and the middle dose of the chloroform fraction have no effect on the propulsion of charcoal meals compared to the negative control group.

### 5.7. *In Vivo* Antidiarrheal Index

The *in vivo* antidiarrheal index (ADI) was measured by considering the delay in defecation (time of onset, *Dfreq*), gut meal travel distance (*Gmeq*), and purging frequency in the number of the wet stools as major parameters. The result of ADI values is presented in Table 5. The greatest ADI was achieved at the dose of 400 mg/kg of crude extract, which is closely related to the positive control group (74.34% vs. 77.65%, respectively). Among the solvent fractions, the highest ADI was recorded at 400 mg/kg of aqueous fraction, which was 62.66%.

## 6. Discussion

Medicinal plants have been employed as a source to develop a novel antidiarrheal agent with advanced efficacy and safety profile. The antidiarrheal drugs derived from plant sources—including crofelemer, from the stem bark latex extract of the *Croton lechleri* tree; berberine, from the extract of the roots and bark of *Berberis aristata*—are effective antidiarrheal drugs for the treatment of secretory diarrhea [47]. Evaluation of the antidiarrheal activity of plants using *in vivo* model is a common practice [46, 48]. Hence, this study aimed to evaluate the antidiarrheal efficacy and the safety of *W. somnifera*. This study supports the safety of the leaf extract of *W. somnifera* as a medicinal plant.

The application of castor oil as a diarrheagenic agent is well known for the evaluation of the antidiarrheal activity of medicinal plants according to previously done experiments [45, 49]. The diarrheagenic effect of castor oil was mediated by its metabolite, ricinoleic acid. After it had been hydrolyzed by the lipase enzyme in the lumen of the intestine, ricinoleic acid caused irritation and inflammation of intestinal epithelial mucus. The irritation and inflammation in turn caused the liberation of endogenous diarrheagenic mediators like nitric oxide, prostaglandin, acetylcholine, histamine, and leukotrienes [50]. Ricinoleic acid binds to and activates prostanoid receptor E4, on the intestinal epithelial mucosa that leads to the activation of adenylyl cyclase. The activation of adenylyl cyclase causes hypersecretion fluid and electrolyte and hypermotility of intestinal smooth muscle that leads to diarrhea [51]. The induction of diarrhea by castor oil is similar to the natural pathophysiology of diarrhea disease. Hence, the use of castor oil in this model was reasonable.

Castor oil-induced diarrheal model was designed to evaluate the overall antidiarrheal activity of the crude extract and the solvent fractions of *W. somnifera* leaves. The crude extract and the solvent fractions displayed a dose-dependent antidiarrheal activity in all diarrheal parameters (the weight of wet stools, the onset of diarrhea, the number of wet stools, and the number of total stools). Medicinal plants with antidiarrheal activity are known for delaying the onset of diarrhea, reducing the weight of diarrheal stool, decreasing the consistency of stool and the number of wet and total stools [52, 53]. Therefore, this finding confirmed that the *W. somnifera* leaves have antidiarrheal activity. Phytochemicals, synthesized as a secondary metabolite, mediate the antidiarrheal activity of the medicinal plant. The antidiarrheal activity of the plants is related to the qualitative and quantitative constitutes of these phytochemicals. The most important phytochemicals having antidiarrheal activity include tannins, alkaloids, glycosides, terpenoids, steroids, phenols, and flavonoids [54, 55]. Flavonoids from *Pistacia integerrima* extract [56] and alkaloids from the seed extract of *Murraya koenigii* demonstrated antidiarrheal activity on castor oil-induced and PGE2-induced diarrhea in rats [57]. The variation in the antidiarrheal efficacy among the doses and the extracts might be due to the qualitative and quantitative difference in the phytochemical constitutes. This castor oil-induced diarrheal model confirmed that the plant extract has antidiarrheal activity; however, it did not explain the antidiarrheal mechanism of action like antisecretory action, enhancing of absorption, and/or antimotility action. Consequently, additional antidiarrheal models such as castor oil-induced enteropooling and castor oil-induced gastrointestinal motility models were developed to suggest the likely antidiarrheal mechanism of action of leaf extracts.

Enteropooling is the accumulation of fluid in the lumen of the intestine. The accumulated fluid is the sum of the fluid that results from the secreted fluid and the fluid already present in the lumen as a result of inhibition of absorption [58]. In castor oil-induced enteropooling, the crude extract of *W. somnifera* (L.) leaves significantly reduced the accumulation of fluid at all doses as confirmed from the drop of both the volume and the weight of intestinal contents. This demonstrated that the antidiarrheal activity of the leaves was a consequence of inhibition of secretion and enhancement of absorption of fluid in the lumen of intestine. This antienteropooling activity contributed to the antidiarrheal activity that was observed in the castor oil-induced diarrhea model. The antienteropooling activity of extract was related to the qualitative and quantitative phytochemical constitutes of the plants. Tannins and flavonoids, which were presented in the extract, exhibited antisecretory activity via inhibition of cystic fibrosis transmembrane conductance regulator protein [59, 60]. Tannins also showed blocking of fluid secretion as a result of denaturing of proteins and by forming a tannate complex coat over the surface of the intestinal mucus which makes the intestinal mucus more resistant to chemical alteration and reduces secretion [61]. Withanolides, extracted from the leaves of *W. somnifera,* revealed COX-2 enzyme, which converts arachidonic acid into prostaglandin, an inhibition that contributed to the antidiarrheal activity of the plant [31]. Prostaglandin induces diarrhea as a result of overstimulation of secretion and inhibition of fluid absorption in the lumen of the gastrointestinal tract. The leaf extract of *W. somnifera* showed anti-inflammatory activity against lipopolysaccharide-induced inflammation [62]. This evidence strengthens the noticed antienteropooling activity of the *W. somnifera* leaves. The observed antienteropooling variations among the doses and extracts could be the difference in the quantities and types of phytochemical constitutes. This antienteropooling activity of the plant was dose dependent. This signified that to produce maximum antienteropooling effect, large-amount dose is required.

In the evaluation of the castor oil-induced gastrointestinal motility model, there was a significant reduction in the movement of charcoal meal marker along the intestinal tract at all tested doses of crude extract. Reduction in the motility of intestinal contents permits an extended contact time at the lumen of the intestine for absorption [63]. This indicates that antidiarrheal activity of castor induces diarrhea model too as a result of the decreasing intestinal motility beyond that of antisecretory activity and empowering of fluid absorption capacity. Both crude and solvent fractions revealed a dose-dependent antimotility activity. The highest antimotility activity was observed at 400 mg/kg dose of the crude. The antimotility activity of the extract was due to the phytochemical constitutes. Flavonoids and terpenoids showed a concentration-dependent inhibition of spontaneous muscle contractions on isolated rat ileum [64]. Alkaloids isolated from the root extract of *Voacanga africana* also showed inhibitory activity on contractility of intestinal muscle against capsaicin-induced contraction of muscle isolated from mouse rectum [65]. Additionally, from a previous study, withanolides, isolated from the leaves of *W. somnifera,* showed significant antispasmodic activity and calcium antagonism action on a spontaneously contracted jejunum isolated from the rabbit intestine [38]. This evidence also strengthens the antimotility activity of the leaves of the plant.

The antidiarrheal index denotes the jointed effects of three diarrheal parameters, namely, purging frequency in the number of wet stools, delay in onset of diarrheal stool, and intestinal motility. The higher the ADI value, the greater the effectiveness of the extract in the treatment of diarrhea [66]. The highest ADI value was produced by the crude extract at its high dose which is directly related to its efficacy in treating diarrhea.

## 7. Conclusions

The finding of this experiment revealed that both the crude extract and the solvent fractions of *W. somnifera* leaves have antidiarrheal activity. Accordingly, this study strengthens the folklore use of the *W. somnifera* leaves for the treatment of diarrhea. However, they require further investigations to be converted to a leading compound.

## Figures and Tables

**Table 1:** Preliminary phytochemical screening result of crude extract and solvent fractions of *Withania somnifera* leaves.

Screened phytochemicals	Crude extract	AF	*n*-BF	CF
Flavonoids	+	+	+	+
Phenols	+	+	+	−
Steroids	+	+	−	+
Terpenoids	+	−	+	+
Tannins	+	+	+	−
Saponins	+	+	−	−
Alkaloids	+	+	+	−
Glycosides	−	−	−	−
Anthraquinones	−	−	−	−

n-BF: n-butanol fraction, AF: aqueous fraction, CF: chloroform fraction, +: present, −: absent.

**Table 2:** Antidiarrheal effects of crude extract and solvent fractions of the *Withania somnifera* leaves on castor oil-induced diarrheal model in mice.

Dose (mg/kg)	OSD (min)	No WS	TNS	WWS (g)	% DI
NC	65.50 ± 4.54	8.33 ± 0.33	9.50 ± 0.56	0.48 ± 0.02	—
Lop	126.67 ± 3.14^a3^	1.5 ± 00.31^a3^	3.50 ± 0.34^a3^	0.21 ± 0.01^a3^	82.35
100CE	79.33 ± 2.95^b3c3^	6.00 ± 0.37^a2b3c3^	6.67 ± 0.34^a2b3c3^	0.36 ± 0.01^a2b3c3^	29.41
200CE	102.00 ± 4.07^a2b3c2^	3.67 ± 0.33^a3b2c1^	4.67 ± 0.33^a3^	0.29 ± 0.02^a3^	56.86
400CE	122.83 ± 3.57^a3 b3c3^	1.67 ± 0.33^a3^	3.17 ± 0.40^a3^	0.27 ± 0.05^b3c3^	80.39
100CF	72.17 ± 3.96^b3c3^	7.87 ± 0.54^b3c3^	10.33 ± 0.52^b3c3^	0.42 ± 0.02^b3c3^	7.8
200CF	80.17 ± 2.17^b3c3^	6.83 ± 0.48^b3c3^	7.83 ± 0.33^b3c3^	0.37 ± 0.02^b3c3^	19.61
400CF	94.50 ± 3.21^a2b3c3^	4.67 ± 0.48^a3b2c2^	6.17 ± 0.54^a3b1^	0.28 ± 0.02^a3b3c3^	45.10
100BF	75.50 ± 3.14^b3c3^	7.00 ± 0.40^b3c3^	8.17 ± 0.40^b3c3^	0.39 ± 0.01^b3c3b2^	17.65
200BF	87.17 ± 3.95^a2b3c3^	6.17 ± 0.21^a2b3c3^	6.67 ± 0.42^a1b3c3^	0.35 ± 0.01^a2b3c3^	27.41
400BF	10.83 ± 2.29^a3b1c1^	3.33 ± 0.42^a3^	4.50 ± 0.62^a3^	0.27 ± 0.01^a2^	60.78
100AF	76.00 ± 4.16^b3c3^	6.50 ± 0.31^a1b3c3^	7.83 ± 0.52^a1b3c3^	0.38 ± 0.01^a1b3c2^	23.53
200AF	98.83 ± 3.86^a2b3c2^	4.17 ± 0.43^a3b3c2^	5.33 ± 0.33^a3b2^	0.32 ± 0.02^a3b2^	50.98
400AF	113.83 ± 2.29^a3^	3.17 ± 0.33^a3^	4.17 ± 0.40^a3^	0.25 ± 0.01^a3^	62.74

The result is expressed as mean ± standard error of the mean; (*n* = 6). ^a^compared to negative control, ^b^negative compared to 3 mg/kg loperamide, ^c^compared to 400 mg/kg crude extract; ^1^*p* < 0.05, ^2^*p* < 0.01, ^3^*p* < 0.00. OSD: onset of diarrhea, No WS: number of wet stools, WWS: weight of wet stools, % DI: percentage of diarrhea inhibition, NC: negative controls (10 ml/kg of 2% Tween 80 in distilled water), CE: crude extract, Lop: 3 mg/kg loperamide, CF: chloroform fraction; BF: n-butanol fraction, AF: aqueous fraction.

**Table 3:** Effect of crude extract and solvent fractions of the *Withania somnifera* leaves on castor oil-induced enteropooling

Dose (mg/kg)	MVIC (g)	% inhibition	MWIC (ml)	% inhibition
NC	0.70 ± 0 0.01	—	0.78 ± 0.04	—
Lop	0.29 ± 0.01^a3^	59.14	0.34 ± 0.01^a3^	56.83
100CE	0.54 ± 0.02^a1b3c3^	23.04	0.58 ± 0.02^a1b3c3^	25.64
200CE	0.39 ± 0.01^a3b1^	44.42	0.42 ± 0.05^a3b1^	45.94
400CE	0.30 ± 0.01^a3^	57.25	0.35 ± 0.02^a3^	54.91
100CF	0.67 ± 0.01^b3c3^	4.52	0.72 ± 0.01^b3c3^	7.69
200 CF	0.63 ± 0.01^b3c3^	9.75	0.71 ± 0.01^b3c3^	9.40
400CF	0.47 ± 0.01^a3b3c3^	33.02	0.52 ± 0.02^a3b3c3^	33.33
100BF	0.65 ± 0.03^b3c3^	7.13	0.69 ± 0.01^b3c3^	11.32
200BF	0.46 ± 0.02^a2b3c3^	34.44	0.51 ± 0.02^a2b3c3^	34.62
400BF	0.37 ± 0.01^a3b2c1^	47.27	0.44 ± 0.01^a3b1^	46.15
100AF	0.60 ± 0.01^b3c3^	14.49	0.64 ± 0.01^b3c3^	37.18
200AF	0.42 ± 0.04^a3b3c3^	40.15	0.43 ± 0.03^a3b2c2^	44.87
400AF	0.34 ± 0.02^a3^	51.55	0.39 ± 0.02^a3^	49.58

The result is expressed as mean ± standard error of the mean; *n* = 6. ^a^compared to negative control, ^b^negative compared to 3 mg/kg loperamide, ^c^compared to 400 mg/kg crude extract; ^1^*p* < 0.05, ^2^*p* < 0.01, ^3^*p* < 0.001. MVIC: mean volume of intestinal content, MWIC: mean weight of intestinal content, NC: negative control (10 ml/kg of 2% Tween 80 in distilled water), CE: crude extract, Lop: 3 mg/kg loperamide, CF: chloroform fraction, BF: n-butanol fraction, AF: aqueous fraction.

**Table 4:** Effects of crude extract and solvent fractions of *Withania somnifera* leaves on castor oil-induced gastrointestinal motility in mice.

Dose given (mg/kg)	LSI (cm)	DTCM (cm)	PI	% inhibition
NC	58.5834 ± 0.32	44.4934 ± 0.85	75.9834 ± 1.75	—
Lop	56.5834 ± 0.94	16.8034 ± 1.22 ^a3^	29.7134 ± 2.13^a3^	60.89
100 CE	58.6134 ± 0.43	35.5234 ± 0.90^a3b2c3^	61.3634 ± 1.71^a2b3c3^	19.23
200 CE	58.2534 ± 0.52	25.3034 ± 1.60^a3b2c2^	43.4634 ± 2.81^a3b2c1^	42.8
400 CE	55.6834 ± 1.08	17.5534 ± 1.64^a3^	31.613.12^a3^	58.39
100CF	58.0534 ± 0.98	44.4234 ± 1.11^b3c3^	76.50.70^b3c3^	7.84
200CF	58.7234 ± 0.64	39.2234 ± 0.57^b3c3^	66.8734 ± 0.95^b3c3^	11.98
400CF	59.2234 ± 0.50	31.5934 ± 1.41^a3b3c3^	53.3234 ± 2.21^a3b3c3^	29.82
100BF	58.6534 ± 0.45	39.4834 ± 1.10 ^b3c3^	67.3234 ± 1.53^b2c3^	11.39
200BF	58.4934 ± 0.52	32.9734 ± 0.94^a3b3c3^	56.3834 ± 1.63^a3b3c2^	25.79
400BF	58.1634 ± 0.83	25.7734 ± 0.76 ^a3b3C2^	44.4234 ± 1.84^a3b2c1^	41.54
100AF	57.8934 ± 0.75	36.9034 ± 0.7^b3c3^	64.7034 ± 1.06^b3c3^	14.85
200AF	58.3434 ± 0.38	27.3134 ± 1.06^a3b3c3^	46.8634 ± 1.96^a3b3c3^	38.32
400AF	58.4834 ± 0.47	20.8434 ± 0.97^a3^	35.6034 ± 1.44^a3^	53.14

The results are expressed as mean ± standard error of mean (*n* = 6). ^a^compared to negative control, ^b^compared to positive control, ^c^compared to 400 mg/kg. NC: negative controls (10 ml/kg 2% Tween 80 in distilled water), CE: crude extract, Lop: 3 mg/kg loperamide, CF: chloroform fraction, BF: n-butanol fraction, AF: aqueous fraction, LSI: length of small intestine, DTCM: distance traveled by charcoal meal, PI: peristalsis index. ^1^*p* < 0.05, ^2^*p* < 0.01, ^3^*p* < 0.001.

**Table 5:** *In vivo* antidiarrheal index of the crude extract and solvent fractions of the *Withania somnifera* leaves in mice.

Dose (mg/kg)	Delay in defecation (onset of time in min)	Gut meal travel distance (*Gmeq*)	Purging frequency (%) (in number of wet stools)	*In vivo* antidiarrheal index (ADI)
NC	—	—	—	—
Lop	93.39	60.89	82.35	77.65
100CE	21.11	19.23	29.41	22.86
200CE	55.73	42.8	56.86	32.9
400CE	87.53	58.39	80.39	74.34
100CF	10.18	7.84	7.84	8.55
200CF	22.40	11.98	19.61	17.39
400CF	44.27	29.82	45.1	39.05
100BF	15.27	11.39	17.65	14.53
200BF	33.08	25.79	27.41	28.6
400BF	69.21	41.54	60.78	55.91
100AF	16.03	14.85	23.53	17.76
200AF	50.89	38.32	50.98	46.32
400AF	73.79	53.14	62.74	62.66

NC: negative controls, CE: crude extract, Lop: 3 mg/ml loperamide, CF: chloroform fraction, BF: n-butanol fraction, AF: aqueous fraction.

## Data Availability

All the datasets used and/or analyzed during the study are available from the corresponding author on reasonable request.
